# Tregs Promote the Differentiation of Th17 Cells in Silica-Induced Lung Fibrosis in Mice

**DOI:** 10.1371/journal.pone.0037286

**Published:** 2012-05-15

**Authors:** Laiyu Song, Dong Weng, Fangwei Liu, Ying Chen, Cuiying Li, Lei Dong, Wen Tang, Jie Chen

**Affiliations:** Division of Pneumoconiosis, School of Public Health, China Medical University, Shenyang, People's Republic of China; University of Southern California, United States of America

## Abstract

**Background:**

Silicosis is an occupational lung disease caused by inhalation of silica dust and characterized by lung inflammation and fibrosis. Previous study showed that Tregs regulate the process of silicosis by modulating the maintenance of immune homeostasis in the lung. Th17 cells share reciprocal developmental pathway with Tregs and play a pivotal role in the immunopathogenesis of many lung diseases by recruiting and activating neutrophils, but the regulatory function of Tregs on Th17 response in silica induced lung fibrosis remains to be explored.

**Methodology/Principal Findings:**

To evaluate the role of Th17 and IL-17 in the development of silicosis and their interaction with Tregs, Treg-depleted mice model was generated and exposed to silica to establish experimental model of silica-induced lung fibrosis. Here we showed that silica increased Th17 response in lung fibrosis. Tregs depletion enhanced the neutrophils accumulation and attenuated Th17 response in silica induced lung fibrosis. Both mRNA and protein results showed that Tregs exerted its modulatory function on Th17 cells and IL-17 by regulating TGF-β1 and IL-1β.

**Conclusion/Significance:**

Our study suggested that Tregs could promote Th17 cells differentiation by regulating TGF-β1 and IL-1β in silica induced lung fibrosis of mice, which further the understanding of the progress of silicosis and provide a new insight in the regulatory mechanism of Th17 by Tregs in lung inflammation.

## Introduction

Inhalation of silica particle caused silicosis, which is characterized by lung inflammation and fibrosis [1], [2]. Pathogenesis of silicosis involves uncontrolled immune processes [3].

CD4+CD25+ T cells, termed regulatory T cells, which are a stable lineage of cells that plays a suppressive function in the maintenance of immunological tolerance and immune homeostasis, but whose role in protective immunity is not fully understood [4]. In our previous study, we reported a crucial role of Tregs in silicosis that depletion of Tregs could attenuate the progress of silica-induced lung fibrosis [5]. Reduction in the frequency and function of Tregs was found in silicosis patients [6]. These suggest that Tregs play an important role in the development of silicosis.

Th17 lymphocytes, which produced IL-17A (also termed IL-17), IL-17F, and IL-22, represent a recently identified Th cell lineage that plays crucial roles by recruiting neutrophils and other cytokines in lung inflammations and diseases [7], [8]. The differentiation of Th17 cells requires TGF-β, IL-6, and/or IL-23 [9]. The transcription factor retinoic acid-receptor–related orphan nuclear receptor γt (RORγt) mediates their lineage commitment [10]. Th17 lymphocytes are reported to mediate early lung inflammation in experimental silicosis [11]. Th17 cells and Tregs are thought to promote and suppress inflammatory responses, respectively. Tregs have the paradoxical ability to inhibit or promote Th17 response. Some researchers believe that the proliferation of Th17 cells is inhibited by either Tregs or other type of T cells [12], [13]. Other researchers report that Tregs can promote the differentiation of Th17 cells [14]–[16]. The underlying mechanism and possible roles of Tregs in the context of differentiating Th17 cells in silicosis are unclear.

In this study, we used anti-CD25 antibodies to neutralize Tregs continuously and assessed the immune responses of silica-induced lung fibrosis. The objective of this study: (1) to identify the role of Th17 response in silicosis; (2) to elucidate mechanisms of the interactions between Tregs and Th17 cells in experimental silicosis. Depletion of Tregs led to attenuated Th17 response in silicosis which suggested that Tregs could promote acute Th17 response and this function might depend on TGF-β1 and IL-1β.

## Results

### Tregs depletion enhanced neutrophilic inflammation in silica induced lung fibrosis of mice

First we injected anti-CD25 mAb PC61 to generate CD25+ T cell-depleted C57BL/6 mice. Then we examined lung responses to silica in mice injected with saline, silica and silica + anti-CD25 mAb at day 3, 7, 28 and 56. We tested the percentage of CD4+CD25+ Tregs in the spleen by flow cytometer to make sure the successful depletion of CD4+CD25+ Tregs. Injection of anti-CD25 mAb successfully depleted CD4+CD25+Tregs (Figure S1A and 1B). Foxp3 and CTLA-4, the functional phenotype of Tregs, expressed on CD4+CD25+T cells in spleen, HLN and BALF were assessed by flow cytometer (Figure S2 A–F). Injection of anti-CD25 Abs neutralized most of the Tregs and invalided Tregs' function.

**Figure 1:**
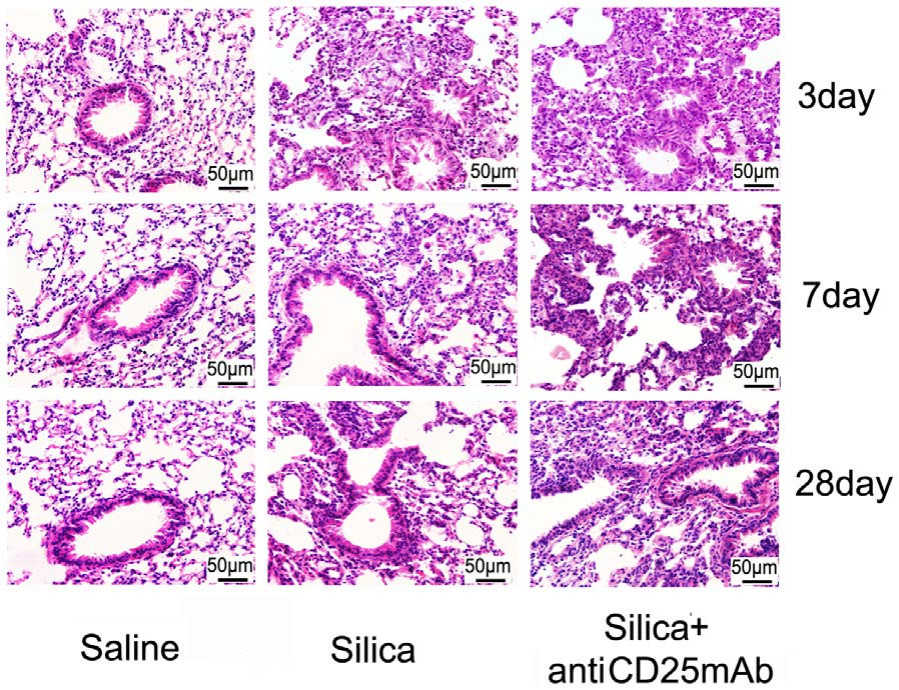
Histopathology changes in mouse lungs after instillation with HE staining (×200). The scale on the graph above was 50 µm; date was day3, day7, day28. Lung sections were stained with H&E. The degree of inflammation was assessed by the histological analysis of six random fields per sample (with n = 5 mice per group).

**Figure 2:**
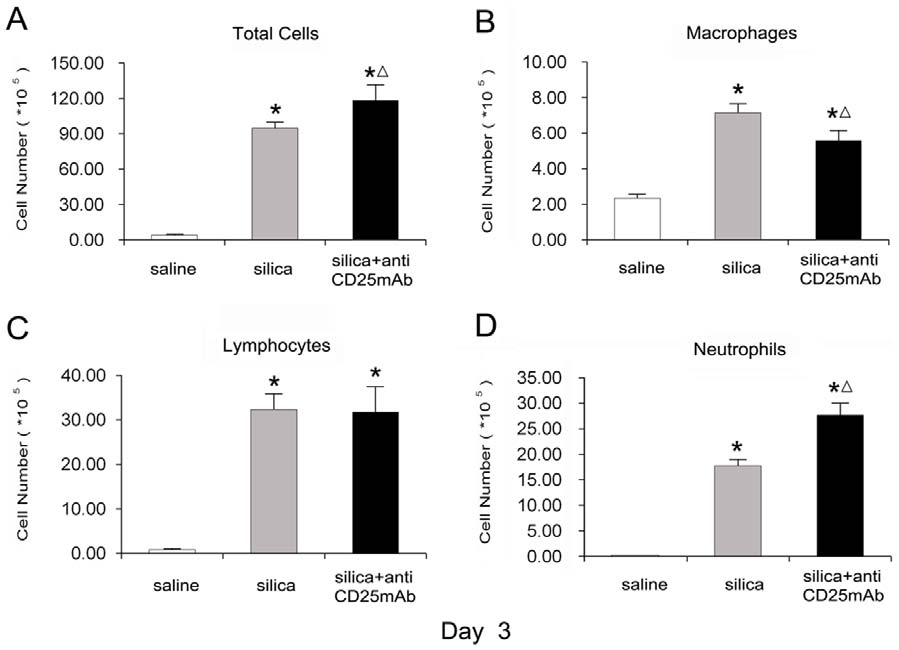
Depletion of Tregs increased the accumulation of inflammation cells in the lung of experimental silicosis mice. The total cells (A), macrophages (B) lymphocytes (C) and neutrophils (D), in BALF(day 3) were counted by using Giemsa staining. Results (n = 5) are shown as mean±SEM (one-way analysis of variance followed by pair-wise comparison with the Student-Newman-Keuls test. *, as compare with the saline control group, P<0.05; Δ, as compared with the silica group, P<0.05).

The inflammation around the bronchioles was increased in Treg-depleted mice compared with silica-treated animals. No obvious abnormalities were observed in the lungs of mice that received saline (Fig. 1) (table 1). The inflammation was graded as following criteria: 0, no cell infiltration and alveolar change; I, minimal cell infiltration and alveolar wall thickening; II, slight cell infiltration and alveolar wall thickening; III, moderate cell infiltration and alveolar wall thickening; IV, severe cell infiltration and alveolar wall thickening. Considering the severer inflammation and infiltrated cells in Treg-depleted group, we examined the accumulation of inflammatory cells (including total cells, macrophages, lymphocytes and neutrophils) in BALF. We found that more inflammatory cells infiltrated in silica-treated and Treg-depleted groups compared with saline control group (Fig. 2A). The number of macrophages reduced clearly in Treg-depleted mice compared with silica-treated mice at day 3 (Fig. 2B). There was no difference in the number of lymphocytes between silica-treated and Treg-depleted groups (Fig. 2C). However, Treg-depleted mice dramatically enhanced neutrophils accumulation compared with silica-treated mice at day 3 (Fig. 2D). These results suggested that depletion of Tregs enhanced neutrophilic inflammation in the early stage of silica induced lung fibrosis.

**Table 1:** Cell infiltration and alveolar change of the mice lungs in each group at day 3, 7, 28 and 56.

Groups	3 day after instillation	7 day after instillation	28 day after instillation	56 day after instillation
**saline control**	0	0	0	0
**Silica**	I	I+∼II	I∼I+	0∼I
**Silica+antiCD25mAb**	I+∼II	II+∼III+	I+∼II	0∼I

The degree of inflammation was assessed by the histological analysis of six random fields per sample (n = 5).

### Depletion of Tregs led to decreased Th17 response in silica induced lung fibrosis of mice

**Figure 3:**
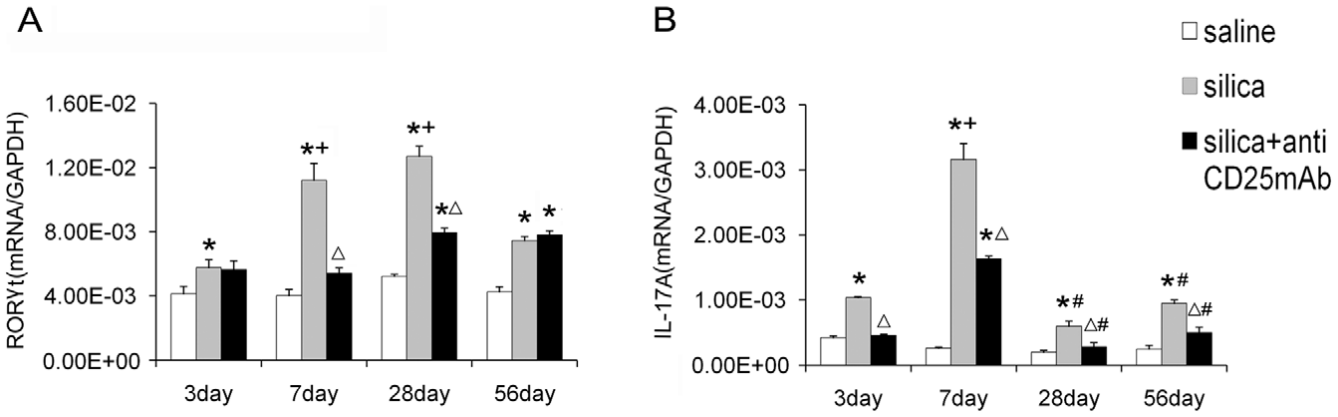
Depletion of Tregs decreased Th17 response in silica induced lung fibrosis. The RORγ-t (A) and IL-17A (B) mRNA were assayed by realtime RT-PCR by using −ΔΔCt method. Results (n = 5) are shown as mean±SEM (one-way analysis of variance followed by pair-wise comparison with the Student-Newman-Keuls test. *, as compare with the saline control group, P<0.05; Δ, as compared with the silica group, P<0.05; + compared with 3day of the same group, P<0.05; #, as compared with 7day of the same group, P<0.05).

To testify the role of Th17 in the inflammation of silicosis, we extracted total RNA from lung homogenates and tested RORγt by real-time RT-PCR. RORγt is a key transforming growth factor of Th17 cells. The mRNA expression of RORγt in silica-treated group was higher compared with saline control group at all the time points, especially at day 7 and day 28 (Fig. 3A). IL-17A, the major effecter cytokine of Th17 cells and usually recognized as IL-17, also increased greatly in silica-treated group and mounted to the highest level at day 3 and 7 (Fig. 3B). Instead, the expression of RORγt in Treg-depleted group reduced significantly compared with that in silica-treated group at day 7 and day 28 (Fig. 3A). IL-17A mRNA decreased significantly in Treg-depleted group compared with that in silica-treated group from day 3 (Fig. 3B). Depletion of Tregs inhibited Th17 response in silica induced lung fibrosis, which suggested that Th17 and IL-17 were not responsible for the severer neutrophilic inflammation in Treg-depleted group.

**Figure 4:**
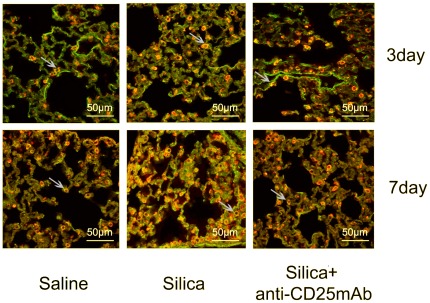
The localization of CD4+IL-17A+ cells in lung tissue examined by immunofluorescence (×600). The CD4+(red) cells, IL-17A+(green) cells and CD4+IL-17A+(yellow) cells were colocalized in the lung tissue sections by confocal immunofluorescence microscope, analyzed with the leica confocal software package. Results from one representative experiment out of 5 (with n = 5 mice per group) are shown.

Next we examined the localization of Th17 cells by immunofluorescence. The CD4+IL-17A+ (yellow) cells were identified in the lung tissue sections by confocal immunofluorescence, data were day 3 and day 7 (Fig. 4). We observed CD4+ T cells (red) infiltrated the whole lung. Some of CD4+ T cells expressed IL-17A (green). But not all the IL-17A+ cells expressed CD4. These results demonstrated that Th17 cells were not the only source of IL-17. NK T cells, γδ T cells and other cells could secrete IL-17 [11], [17]. The number of CD4+IL-17A+ cells in silica-treated group was higher than that of saline control group especially at day 7. There was no significant difference between Treg-depleted group and saline control group at day 3 and day 7. The CD4+IL-17A+ cells in Treg-depleted group was lower than that in silica-treated group at day 7, but no significant difference was observed at day 3 (Fig. 4). These data supported the promotive function of Tregs on Th17 response in experimental model of silica-induced lung fibrosis. Given that the expression of RORγt and IL-17A in Treg-depleted group decreased significantly compared with that in silica-treated group mentioned above, these results suggested that Th17 and IL-17 were not responsible for the accumulation of neutrophils and severer lung inflammation.

### The regulatory function of Tregs on Th17 cells in silica induced lung fibrosis might depend on TGF-β1 and IL-1β

**Figure 5:**
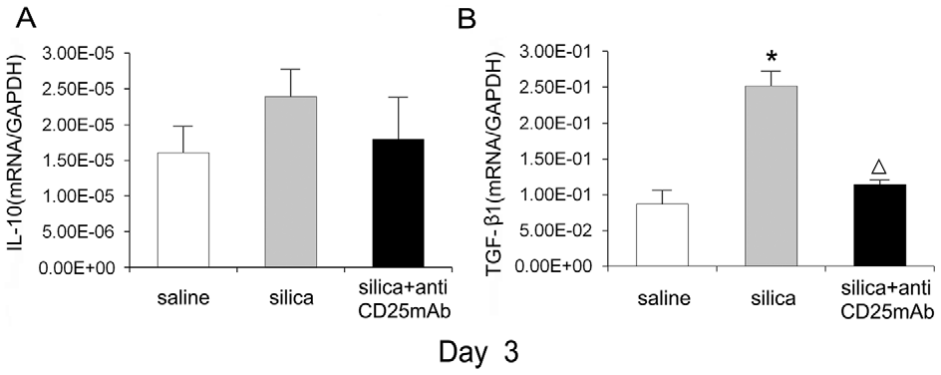
TGF-β1 and IL-10 decreased in the Tregs depletion group and TGF-β1 may contribute to the Th17 cells differentiation. The IL-10 (A) and TGF-β1 (B) mRNA (day 3) were assayed by realtime RT-PCR by using −ΔΔCt method. Results (n = 5) are shown as mean±SEM (one-way analysis of variance followed by pair-wise comparison with the Student-Newman-Keuls test. *, as compared with the saline control group, P<0.05; Δ, as compared with the silica group, P<0.05).

Considering the reduction of Th17 cells in Treg-depleted models, we speculated Tregs played important roles in the differentiation of Th17 cells. We tested the level of IL-10, which could inhibit Th17 [13], and TGF-β, which could promote Th17 differentiation with the co-work of some pro-inflammatory cytokines [9], to insure whether Tregs regulated Th17 response through IL-10 and/or TGF-β1. An increased expression of IL-10 mRNA in lung homogenates from silica-treated mice was observed and depletion of Tregs caused a tendency of reduction of IL-10 expression. There was no statistical difference between three groups at day 3 (Fig. 5A), but the statistical difference could be found at other time points [5]. The level of TGF-β1 mRNA in silica-treated group increased significantly compared with that in saline control group and decreased significantly in Treg-depleted group compared with that in silica-treated group. No significant difference could be found between Treg-depleted group and saline control group at day 3 (Fig. 5B). TGF-β1 mRNA in silica-treated group at other time points increased compared with that in Treg-depleted group [5]. Immunohistochemistry for TGF-β1 demonstrated a similar change that paralleled with the mRNA expression. Positively stained cells in the silica treated group were much higher than those in the saline control group and Treg-depleted group (Figure S3). ELISA for IL-10 and TGF-β1 of lung bronchoalveolar lavage also demonstrated a similar protein level (Figure S5 A and B). All data suggested that TGF-β1 might contribute to the differentiation of Th17 in silicosis.

**Figure 6:**
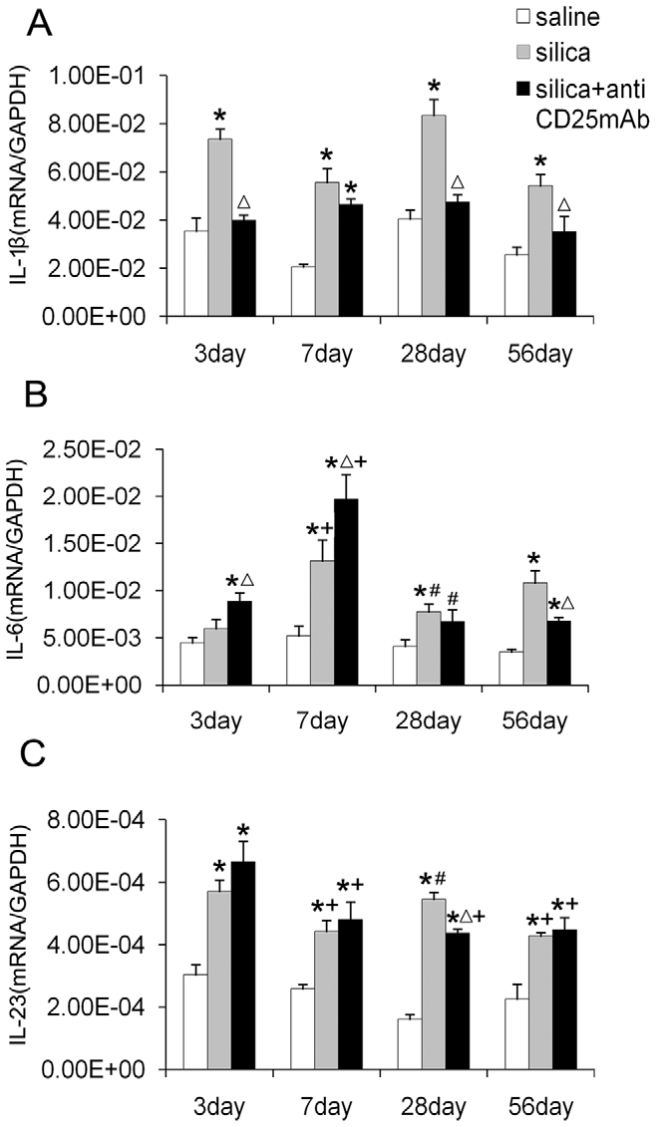
The regulatory function of Tregs on Th17 and IL-17A may depend on IL-1β but not IL-6 and IL-23. The IL-1β (A), IL-6 (B) and IL-23 (C) mRNA were assayed by realtime RT-PCR by using −ΔΔCt method. Results (n = 5) are shown as mean±SEM (one-way analysis of variance followed by pair-wise comparison with the Student-Newman-Keuls test.*, as compared with the saline control group, P<0.05; Δ, as compared with the silica group, P<0.05; +, as compared with 3day of the same group, P<0.05; #, as compared with 7day of the same group, P<0.05).

IL-1β could promote the differentiation of Th17 cells [18]. To investigate whether Tregs manipulate Th17 cells differentiation by regulating IL-1β, we examined the IL-1β mRNA expression in the lung of mice. Both silica-treated and Treg-depleted groups showed higher level of IL-1β expression compared with saline control group. In silica-treated group, the level of IL-1β was significantly higher than that in the saline control group at all the time points. The level of IL-1β in Treg-depleted group decreased significantly compared with that in silica-treated group at day 3, 28 and 56 (Fig. 6A). Immunohistochemistry for IL-1β also demonstrated the differences between three groups that positively stained cells increased in silica-treated and Treg-depleted groups, but more significantly in silica-treated group (Figure S4). All these data suggested that IL-1β secretion increased in response to silica and Tregs depletion decreased the level of IL-1β in silica induced lung fibrosis. This suggested that Tregs modulated the Th17 differentiation in silica-induced lung fibrosis in an IL-1β dependent way.

Then we examined the cytokine IL-6, which could promote the differentiation of Th17 in the presence of TGF-β, and IL-23, which was important in Th17 maintenance and proliferation. Depletion of Tregs led to dramatically increasing expression of IL-6 at day 3 and 7 (Fig. 6B). At day 28 and 56, the level of IL-6 decreased gradually. The IL-6 expression in Treg-depleted group decreased significantly compared with that in silica-treated group at day 28. Although IL-6 expression increased in the absence of Tregs, Th17 differentiation was still inhibited. These data suggested that the pro-inflammatory cytokine IL-6 did not contribute to the decrease of Th17 in Treg-depleted group. The IL-23 expression in Treg-depleted and silica-treated groups increased significantly compared with that in saline control group. IL-23 reached to the highest point at day 3, and then decreased gradually. IL-23 expression in Treg-depleted group was a slightly higher than that in silica-treated group at day 3 and 7 (Fig. 6C), which suggested that IL-23 in silicosis was not regulated by Tregs. In mice model of silica-induced lung fibrosis, IL-6 and IL-23 seemed independent to the modulation of Th17 by Tregs.

**Figure 7:**
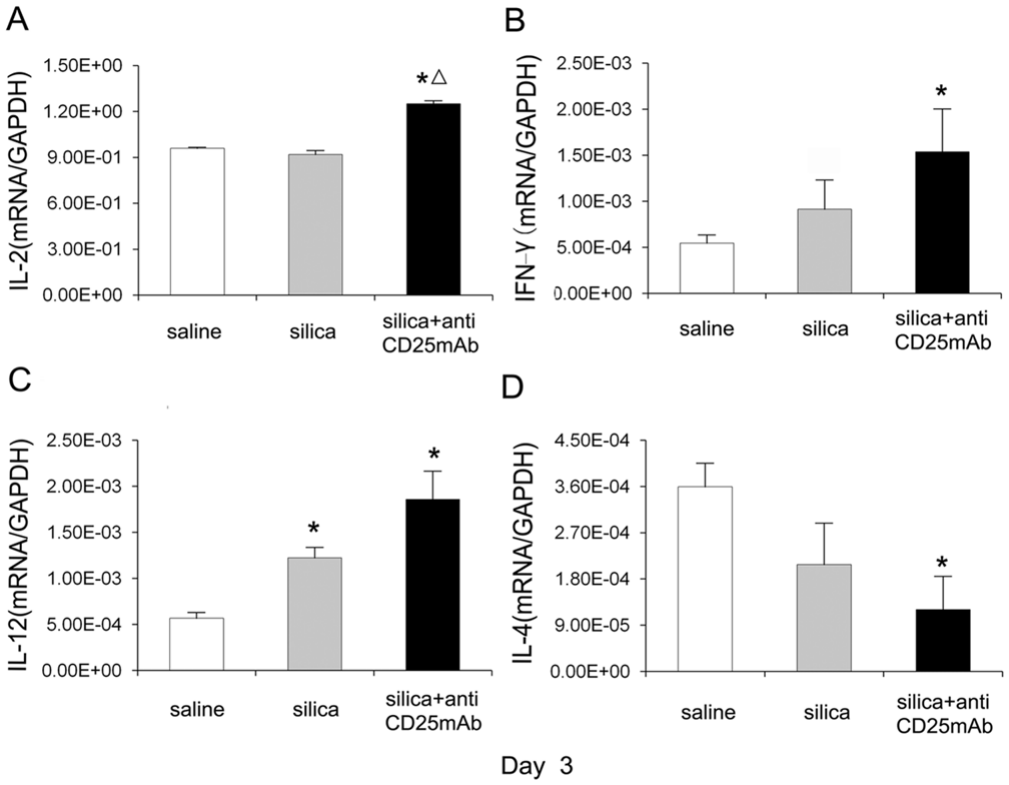
Th1 type of cytokines took the advantage over Th2 cytokine in the depletion of Tregs. Typical Th1 IL-2 (A), IFN-γ (B), IL-12 (C) and Th2 IL-4 (D) cytokines (day 3) were assayed by realtime RT-PCR by using −ΔΔCt method. Results (n = 5) are shown as mean±SEM (one-way analysis of variance followed by pair-wise comparison with the Student-Newman-Keuls test. *, as compared with the saline control group, P<0.05; Δ, as compared with the silica group, P<0.05).

Th1 and Th2 cells were indicated in several studies of lung fibrosis induced by silica particles [19]–[21]. Th immune responses could benefit from the accumulation of neutrophils in silica induced lung inflammation [3]. Therefore we tested IL-2, IFN-γ and IL-12 for Th1 and IL-4 for Th2 to investigate the major type of inflammation which contributes more in the absence of Tregs. At day 3, the expression of IL-2 increased significantly in Treg-depleted group compared with that in silica-treated and saline control groups. There was no difference of IL-2 expression between silica-treated group and saline control group (Fig. 7A). The expression of IFN-γ increased in Treg-depleted and silica-treated groups compared with that in saline control group, but only increased significantly in the Treg-depleted group (Fig. 7B). The IL-12 mRNA increased significantly in both silica-treated and Treg-depleted groups compared with saline control group at day 3 (Fig. 7C). The mRNA of IL-4, which represented Th2 cytokines, decreased significantly in Treg-depleted group (Fig. 7D). ELISA for IFN-γ also showed a higher protein level in silica-treated and Treg-depleted groups, but the difference between silica-treated group and Treg-depleted group was not significant (Figure S5 C). The level of IL-4 in Treg-depleted group was lower than that in silica-treated group (Figure S5 D). These results suggested that Th1 cells and/or Th1 cytokines benefited from the severer neutrophilic inflammation in experimental model of silica-induced lung fibrosis in the absence of Tregs; since the differentiation of Th17 cells could be inhibited by the Th1 cytokine [12], enhancement of Th1 cytokines might inhibit Th17 differentiation and IL-17 secretion.

## Discussion

Silicosis is an occupational lung disease caused by inhalation of silica dust and characterized by lung inflammation and fibrosis. The pathogenesis of silicosis involves alveolar cell injury and activation followed by cytokine signaling and cell recruitment in the areas of silica dust deposition [1], [2]. T lymphocytes play crucial roles in the pathogenesis of silicosis [19]–[21]. Their role and the reciprocal influence between them in the development of pulmonary fibrosis still need to be explored. In this study, we demonstrated that silica could induce Th17 response in lung inflammation and Tregs depletion decreased Th17 differentiation and IL-17 secretion in silica induced lung fibrosis. Tregs might influence Th17 differentiation by regulating TGF-β1 and IL-1β in the mice model of abnormal repair and fibrosis against silica particles.

In this study, we used an established Treg-depleted mice model by injection of the anti-CD25mAb (PC61) to neutralize CD4+CD25+ Tregs [5], [22]. The level of Foxp3, a functional phenotype of Tregs, decreased in Treg-depleted mice; these findings were confirmed by both real-time PCR and flow cytometry as described this article and previously [5]. These results were consistent with that the anti-CD25 mAb selectively depleted those cells expressing the highest levels of CD25, a population recently demonstrated to comprise primarily Foxp3-expressing cells [23], [24]. There might be some CD25-Foxp3+Treg cells and the anti-CD25mAb could not neutralize all the CD4+CD25+ Tregs [25], [26]. However, according to our knowledge in current stage, we thought the administration of anti-CD25mAb remains a commonly method which is used to deplete natural Tregs in vivo [27], [28]. We then examined the inflammation of lung tissues around the bronchioles by light microscope at day 3, 7, 28 and 56. There were more cell infiltration and severer alveolar change in Treg-depleted group compared with silica-treated group at day 3, 7, 28 and 56 [29]. Then we counted the inflammatory cell number in BALF of mice to clarify what kinds of cells were responsible for the severer inflammation in Treg-depleted group. Numerous studies suggested that macrophages, neutrophils and lymphocytes were important for the early lung inflammation in silicosis [21], [30], [31]. However, we found neutrophils but not macrophages or lymphocytes were responsible for the severer inflammation in the early stage of Treg-depleted group. Neutrophils played important roles in the inflammation of silicosis by damaging DNA and lung epithelial cells, which would trigger Th immune responses and be responsible for early lung inflammation in silicosis [3], [32], [33].

Next, we want to study the Th17 response in silica induced lung fibrosis. In a number of models of lung disease, Th17 and/or IL-17 mediate the lung inflammation especially at the early stage by recruiting neutrophils and other cytokines [8], [34]. Some groups have demonstrated that the Th17-Polarized immune response exists in a murine model of lung inflammation [35]. Th17 response was identified in the inflammation of experimental silicosis [11]. Therefore we examined RORγt and IL-17A, the key transforming growth factor of Th17 and the major functional cytokine of Th17 [10]. The expression of RORγt and IL-17A increased significantly during the inflammatory stage in silica induced lung fibrosis, which confirmed other results that Th17 response increased in the silica induced lung fibrosis [11]. But the increasing tendency of RORγt and IL-17A were not the same. The level of RORγt was increased gradually and reached to the highest point at day 28. The level of IL-17A increased dramatically at day 7. This phenomenon might because the IL-17 was not secreted by Th17 alone [17], [36]. The immunofluorescence results showed that the CD4+IL-17A + (yellow) cells in silica-treated group were more than those in the other two groups. Our results testified that Th17 response increased in the inflammation of silica induced lung fibrosis. But the level of RORγt and IL-17A in Treg-depleted group decreased significantly compared with that in silica-treated group. Therefore, Tregs depletion decreased Th17 response in silica induced lung fibrosis. These results suggested that Th17 and IL-17 were not responsible for the severer neutrophilic inflammation in Treg-depleted group, which was also confirmed by the observed decrease of CD4+IL-17A + cells (yellow) and the increase of CD4+ T lymphocytes (red) in the Treg-depleted group examined by immunofluorescence mentioned above.

To investigate the mechanism of how Tregs regulate Th17 differentiation in silicosis, we firstly investigated the cytokine IL-10 and TGF-β1: two crucial functional factors secreted by Tregs and played important roles in Th17 generation [9], [13], [37]. There was no statistical difference of IL-10 mRNA among the three groups at day 3, the difference of IL-10 between three groups also confirmed by ELISA. Considering our earlier work that the inhibitory cytokine IL-10 increased significantly in silica-treated group, we had a reason to believe that Tregs did not manipulate Th17 response in an IL-10 dependent way in silica-induced lung fibrosis. TGF-β1, which was also the major functional cytokine of Tregs [38] and could promote Th17 differentiation [37], might influence Th17 progression in the inflammation of experimental silicosis. Here we showed that TGF-β1 mRNA in Treg-depleted group decreased compared with that in silica-treated group, which might cause the reduction of Th17 and IL-17 in that group. The difference of TGF-β1 between three groups also testified by Immunohistochemistry and ELISA. Therefore Tregs might regulate Th17 differentiation in a TGF-β1 dependent way in experimental model of silica-induced lung fibrosis.

Tregs are required to control exaggerated Th1 and Th2 responses [39]–[45]. Without the regulatory function of Tregs or its cytokines, Th1 cells and/or Th2 cells might expand and benefit from the more severe neutrophilic inflammation in silica induced lung fibrosis [3], [32]. The Th1 cytokines expression increased significantly and Th2 cytokine expression decreased in Treg-depleted group compared with that in silica-treated group at day 3. We have previously reported that Tregs could regulate Th1/Th2 polarization by suppressing Th1 response during the lung inflammation in silica-induced lung fibrosis [5]. Therefore we believed that a typical Th1 response instead of Th17 and Th2 response was predominant in the inflammatory stage of Treg-depleted mice model of silicosis. Many studies had demonstrated the inhibitory function of Th1 cytokines on Th17 response [12], [46]. Our results also suggested that the increased Th1 response might inhibit Th17 response in Tregs depleted mice model of silicosis.

The pivotal function of IL-1β and NALP3 inflammasome in the development of lung fibrosis had been proved before [47]–[50]. Some studies demonstrated that the silica particle engulfed by macrophages would trigger the IL-1β secretion [30]. Some studies have demonstrated the pivotal function of IL-1β in promoting Th17 response [18], [51]–[53]. In this regard, silica particle would induce the Th17 differentiation by promoting IL-1β secretion. The higher IL-1β mRNA expression and protein level in silica-treated group might induce the Th17 inflammation in silica-induced lung fibrosis. Considering the increased number of macrophages in silica-treated group, we have a reason to believe that it might contribute to the Th17 differentiation in a silica-macrophages-IL-1β-Th17 way in experimental model of silica-induced lung fibrosis. The IL-1β level in Treg-depleted group decreased significantly compared with that in silica-treated group, which suggested that Tregs depletion reduced IL-1β secretion in experimental model of silica-induced lung fibrosis. These results suggested that Tregs depletion might reduce Th17 response by decreasing IL-1β secretion. Therefore Tregs might promote Th17 response by modulating the macrophages-IL-1β-Th17 pathway in silica induced lung fibrosis.

Then we examined the inflammatory cytokines IL-6 and IL-23, which would recruite neutrophils and promote Th17 differentiation and maturation [54]–[57]. Our results showed that IL-6 and IL-23 expression in Treg-depleted and silica-treated groups were higher in the early stage, which suggested IL-6 and IL-23 might contribute to the early neutrophilic inflammation of those two groups, especially Treg-depleted groups. And the increasing IL-6 and IL-23 expression in Treg-depleted group were not accompanied by a rising Th17 response in that group. Therefore IL-6 and IL-23 might not be responsible for the reduction of Th17 in experimental silicosis model. These data also suggested that IL-6 and IL-23 might influence Th17 response in a different way from IL-1β in silica induced lung inflammation and fibrosis.

Altogether, our findings suggest that silica could induce Th17 response in lung inflammation and Tregs depletion decreased Th17 differentiation and IL-17 secretion in silica induced lung fibrosis. Tregs may modulate Th17 differentiation by regulating TGF-β1 and IL-1β but not by IL-6 or IL-23. These findings further our understanding of the progress of silicosis and provide a new insight in the regulatory mechanism of Th17 by Tregs in lung inflammation.

## Materials and Methods

### Mice

Healthy female C57BL/6 mice at 6–8 weeks age were purchased from SLAC Laboratory animal co.LTD. (Shanghai, CHINA). All animals were housed in a specific pathogen-free environment and maintained on standard mouse chow with free access to food and water. All animal experiments were approved by the Animal Care and Use Committee at the China Medical University with a permit number of CMU62043013, which complies with the National Institute of Health Guide for the Care and Use of Laboratory Animals.

### Silica preparation

Silica was purchased from Sigma (St., Louse, MO, USA). The content of the SiO_2_ dust was >99%, and the particle size of 80% SiO_2_ dust was between 1 and 5 µm. Silica was grinded in saline for 3 hours, boiled in 1N HCl, washed, dried, suspended in sterile saline. Suspensions were sonicated for 10 min before use.

### Silica exposure

60 mice were randomly divided into three groups (n = 20) as follows: the silica+anti-CD25mAb group, silica group and the saline control group. Animals were anesthetized with intraperitoneal injection of 2% pentobarbital sodium 45 mg/kg body weight. The trachea was exposed by opening the neck skin and blunt dissection. A 7-gauge needle was used to insert into the trachea trans-orally. Mice received either the suspension of 3 mg silica in a total volume of 100 µl sterile saline or just the sterile physiological saline in same volume. The site of surgery was sutured and cleaned with penicillin and the mice were allowed to recover until they were sacrificed.

### CD4+CD25+ regulatory T cell depletion

Mice from silica+anti-CD25mAb group or silica-treated group and saline control group received intraperitoneal injection with 100 µg of anti-CD25 mAb (PC61) (BioLegend, 11080 Roselle Street, San Diego, CA 92121) or rat IgG1 (BioLegend, 11080 Roselle Street, San Diego, CA 92121) in phosphate buffered saline just one day before the silica exposure. And repeatedly treated by i.p. with PC61 100 µg or rat IgG1 of the same volume every 7 days after the silica exposure for continuing depletion.

### Bronchoalveolar lavage and differential cell counts

Mice were sacrificed at 3, 7, 28 or 56 days after challenged by silica instillation or sterile physiological saline. The lungs were removed and washed in cold PBS. Bronchoalveolar lavage fluid (BALF) was obtained by cannulating the trachea, injecting and retrieving 1 ml aliquots of sterile physiological saline for 3 times. The BALF was centrifuged at 1500 rpm for 8 min at 4°C. After lysis of RBC, the BAL cell pellet was washed and resuspended in PBS. The total cell counts were determined using standard hematologic procedures. Cytospin of BAL was prepared and stained with the Wright-Giemsa method. Macrophages, neutrophils or lymphocytes were identified on 200 cells using standard morphologic criteria.

### Pathological examination

Lung was fixed in 4% paraformaldehyde-PBS. The tissue was embedded in paraffin, cut in 6 µm-thick sections. The tissue sections were stained with hematoxylin and eosin (H&E). The inflammation was graded as follows: 0, no cell infiltration and alveolar change; I, minimal cell infiltration and alveolar wall thickening; II, slight cell in filtration and alveolar wall thickening; III, moderate cell infiltration and alveolar wall thickening; IV, severe cell infiltration and alveolar wall thickening.

### Confocal immunofluorescence assay

Following extensively rinsing with 0.01 M PBS (pH 7.4) twice, the slides of lung tissue were blocked with goat serum (Histostain-Plus Kits, ZSBG-BIO) for 30 min to reduce nonspecific binding, then incubated with PECY7-conjugated anti-mouse CD4 antibody 10 µg/ml (BD Pharmingen, San Jose, CA, USA) at 4°C overnight. After washing with PBS for three times, the slides were incubated with Alexa Fluor 488-conjugated anti-mouse IL-17A antibody 10 µg/ml (eBioscience, San Diego, CA 92121, USA) for 2 h at room temperature. The localization of Th17 cells was captured by a confocal laser scanning microscope (TCS sp2/AOBS,LEICA) and analyzed with the leica confocal software package.

### Immunohistochemistry

After dewaxed, the sections were blocked and then incubated overnight at 4°C with antibodies (TGF-β1 sc-146 and IL-1β sc-7884, SANTA CRUZ BIO, United States) at 4°C overnight. After washing with PBS for three times, the slides were incubated with second antibody (Histostain-Plus Kits 9001, ZSBG-BIO) and incubated at 37°C for 30 min. The slides were captured by a upright microscope (Eclipse 80i, Nikon) and analyzed with the NIS-Elements software package.

### ELISA assay of cytokines in BALF

The ELISA plate was coated with 100 µl capture antibody in coating buffer per well of ELISA kit (eBioscience, San Diego, CA 92121, USA) and incubated overnight at 4°C. The plate was washed with 250 µl wash buffer. Then the well was blocked with 200 µl assay diluent, incubated 1 h at room temperature (RT). A volume of 100 µl BALF or the different dilutions of standard (for standard curve) were added to each well, incubated 2 h at RT. The well was incubated with 100 µl detection antibody 1 h at RT, followed by incubating with 100 µl avidin-HRP 30 min at RT. Hundred microliters of substrate solution was added to each well to incubate 15 min at RT. Add 50 µl of Stop Solution to stop the reaction. The plate was read at 450 nm and analyzed. The ELISA was performed in triplicate.

### Purification of hilar lymph nodes and spleen cells

The hilar lymph nodes (HLN) were harvested and dissected by using needles then digested with 0.25% trypsin for 5 min at 37°C. 3% fetal bovine serum-PBS was used to end the digestion. Centrifuge it at 1500 rpm for 8 min at 4°C. The HLN cell pellet was washed and resuspended in PBS. The spleens were removed, grinded and mechanically dissociated in cold PBS. After lysis of RBC, spleen cells were washed and resuspended in PBS.

### Flow cytometry

Analysis of cell surface marker expression was performed using a FACSCantoII (BD, Franklin Lakes, NJ USA) system. Briefly, the cells from BALF, HLN and spleen were re-suspended in PBS and blocked with purified rat anti-mouse CD16/CD32 (BD Pharmingen, San Jose, CA, USA) for 10 min at 4°C. Cells were then incubated with anti-mouse PerCP-conjugated CD3 (BD Pharmingen, San Jose, CA, USA), anti-mouse PE-Cy7-conjugated CD4 (BD Pharmingen, San Jose, CA, USA), and anti-mouse APC-conjugated CD25 (BD Pharmingen, San Jose, CA, USA) for 20 min at 4°C in the dark. After cellular surface staining, cells were washed twice with 3% FBS-PBS. 1 ml working solution (0.25 ml fixation/permeabilization concentrate and 0.75 ml fixation/permeabilization diluent) (eBioscience, San Diego, CA 92121, USA))was added to fix and permeate the cell membrane for 30 min at 4°C in the dark. To label the nuclear factor Foxp3, cells were incubated with anti-mouse FITC-conjugated Foxp3 (eBioscience, San Diego, CA 92121, USA) for 1 h at 4°C in the dark. In addition, cells from HLN and spleen were incubated with anti-mouse PE -conjugated CTLA-4 (BD Pharmingen, San Jose, CA, USA) for 1 h at 4°C in the dark. After then, cells were washed twice with 3% FBS-PBS and re-suspended in 1% paraformaldehyde-PBS. Dead cells were gated out depending on forward scattering (FSC) and side scattering (SSC). Cells were analyzed with Diva software.

### RNA extraction and realtime RT-PCR

Total RNA was isolated from lung homogenates using the TRIZOL® Reagent (Invitrogen, Carlsbad, CA, USA) according to the manufacturer's protocol. The RNA concentration and the ratio of A 260/280 of were determined by UV spectrophotometer. 2 µg total lung RNA of each animal from each treatment group at each time point was reverse transcribed in a volume of 20 µl using the following program: 37°C for 15 min and 85°C for 5 s. For IL-12, IL-23 and RORγt, reversed cDNA was dectected by SYBER Green technology on an ABI7500 system (Applied Biosystems) according to the manufacturer's instruction using the following primers: IL-23 sense 5′-ACATGCACCAGCGGGACATA-3′; anti-sense 5′- CTTTGAAGATGTCAGAGTCAAGCAG-3′; IL-12 sense 5′- TGTCTTAGCCAGTCCCGAAACC-3′; anti-sense 5′-TCTTCATGATCGATGTCTTCAGCAG-3′; RORγt sense 5′-ACGGCCCTGGTTCTCATCA-3′; anti-sense 5′- CCAAATTGTATTGCAGATGTTCCAC-3′. The primers and the Taqman probes for several other genes were designed with the Primer 3 (http://frodo.wi.mit.edu/primer3) and the sequences were blasted (http://www.ncbi.nlm.gov/BLAST/). Primer sequences were as follows: IL-2, sense 5′-TTGAGTGCCAATTCGATGATGAG-3′, antisense 5′-TTGAGATGATGCTTTGACAGAA GG-3′; IFN-γ, sense 5′- AAGCGTCATTGAATCACACCTG -3′, antisense 5′-TGACCTCAAACTTGGCAATACTC-3′; IL-4, sense 5′-AAAATCACTTGAGAGAGATCATCGG-3′, antisense 5′-GTTGCTGTGAGGACGTTTG G-3′; IL-10, sense 5′-GGGGCCAGTACAGC CGGGAA-3′, anti-sense 5′-CTGGCTGAAGG CAGTCCGCA-3′; TGF-β1, sense 5′-TGTG GAACTCTACCAGAAATATAGC-3′, anti-sense 5′-GAAAGCCCTGTATTCCGTCTC-3′; IL-17A, sense5′-GCAAAAGTGAGCTCCAGAAGG-3′ anti-sense 5′- TCTTCATTGCGGT GGAGAGTC-3′ IL-1β, sense 5′-TGACCTGGGCTGTCCTGATG-3′, anti-sense, 5′-GG TGCTCATGTCCTCATCCTG-3′ IL-6, sense 5′-CAATTCCAGAAACCGCATGAAG-3′, anti-sense 5′-GTAGGGAAGGCCGTGGTTG-3′; GAPDH, sense5′-CAATGTGTCCGTCGTGGATCT-3′, anti-sense 5′-GTCCTCAGTGTAGCCCAAGATG-3′; The probe sequences were as follows: IL-2, 5′-(FAM) CCTCAGAAAGTCCACCACAGTTGCT (BHQ1)-3′; IFN-γ, 5′-(FAM) CTTCTTCAGCAACAGCAAGGCGAA (BHQ1)-3′; IL-4, 5′-(FAM) TGGCGTCCCTTCTCCTGTGACCTCG (BHQ1)-3′; IL-10, 5′-(FAM) GCACCCACTTCCCAGTCGGCCAGAGCC (BHQ1)-3′; TGF-β1, 5′-(FAM) TTCAGCCACTGCCGTACAACTCCAG (BHQ1)-3′; IL-17A, 5′-(FAM) CCTCAGACTACCTCAACCGTTCCAC (BHQ1)-3′; IL-1β, 5′-(FAM) TCGCAGCAGCACATCAACAAGAGC (BHQ1)-3′; IL-6, 5′-(FAM)CACCAGCATCAGTCCCAA GAAGGCA(BHQ1)-3′; GAPDH, 5′-(FAM) CGTGCCGCCTGGAGAAACCTGCC (BHQ1)-3′. 2 µl cDNA was used in a 25 µl realtime PCR reaction volume. The difference of the amplification efficiency between the target gene and the housekeeping gene was identified by compared the slopes of the standard curves. The PCR reactions were run on ABI 7500 (Applied Biosystems) using the following program: (1)SYBER Green: 95°C for 30 s, and 40 cycles of 95°C for 5 s and 60°C for 34 s;(2)Taq man: 95°C for 30 s, and 40 cycles of 95°C for 5 s and 60°C for 34 s. Analysis was performed using the 7500 system software (Applied Biosystems).

### Statistical analysis

The SPSS 16.0 software was used to conduct statistical analyses. The differences between values were evaluated through a one-way analysis of variance (ANOVA) followed by pair-wise comparison with the Student-Newman-Keuls test. P<0.05 was considered statistically significant and all values were means ±SEM.

## Supporting Information

Figure S1 **Injection of anti-CD25 mAb sufficiently depleted CD4^+^CD25^+^ regulatory T cells in vivo continuously.** (A) C57BL/6 mice were treated i.p. with 100 µg anti-CD25 mAb or control IgG, the percentage of CD4^+^CD25^+^ Treg cells in the spleen was assayed by using anti-CD4 and CD25 mAb by flow cytometry (day 3). Results (n = 5) are shown as mean±SEM. (B) Percentage of CD4^+^ T cells expressing CD25 was shown in the graph (day 3). Results (n = 5) are shown as mean±SEM (one-way analysis of variance followed by pair-wise comparison with the Student-Newman-Keuls test. *, as compared with the saline control group, P<0.05; Δ, as compared with the silica group, P<0.05).
(TIF)

Figure S2 **FOXP3 and CTLA-4, functional markers of Tregs, reduced clearly with the depletion of CD4+CD25+ Tregs.** FOXP3+ Tregs in spleen, HLN, and BALF were calculated by flow cytometry (day 3) (A). The percentage of FOXP3+ Tregs was shown in the graph (B, spleen; C, HLN; D, BALF). The percentage of CTLA-4+ Tregs was shown in the graph (E, spleen; F, HLN). Results (n = 5) are shown as mean±SEM. (one-way analysis of variance followed by pair-wise comparison with the Student-Newman-Keuls test. *, as compared with the saline control group, P<0.05; Δ, as compared with the silica group, P<0.05).
(TIF)

Figure S3 **The protein level of TGF-β1 in mouse lungs examined by Immunohistochemistry (×200).** Lung sections were examined by Immunohistochemistry. Positive staining is yellow brown, six random fields per sample (with n = 5 mice per group).
(TIF)

Figure S4 **The protein level of IL-1β in mouse lungs examined by Immunohistochemistry (×200).** Lung sections were examined by Immunohistochemistry. Positive staining is yellow brown, six random fields per sample (with n = 5 mice per group).
(TIF)

Figure S5 **Treg cells affected the BALF cytokines in the mice model of silica-induced lung inammation.** IL-10 (A), TGF-β1(B), Typical Th1 (IFN-γ)(C) and Th2 (IL-4)(D) cytokines in BALF were assayed by ELISA. (n = 3) (one-way analysis of variance followed by pair-wise comparison with the Student-Newman-Keuls test. *, as compared with the saline control group, P<0.05; Δ, as compared with the silica group, P<0.05).
(TIF)
