# Supplementary figures and images for: Tregs Promote the Differentiation of Th17 Cells in Silica-Induced Lung Fibrosis in Mice
Source: PLoS One. 2012 May 15;7(5):e37286. doi: 10.1371/journal.pone.0037286 (PMC3352873; doi:10.1371/journal.pone.0037286)

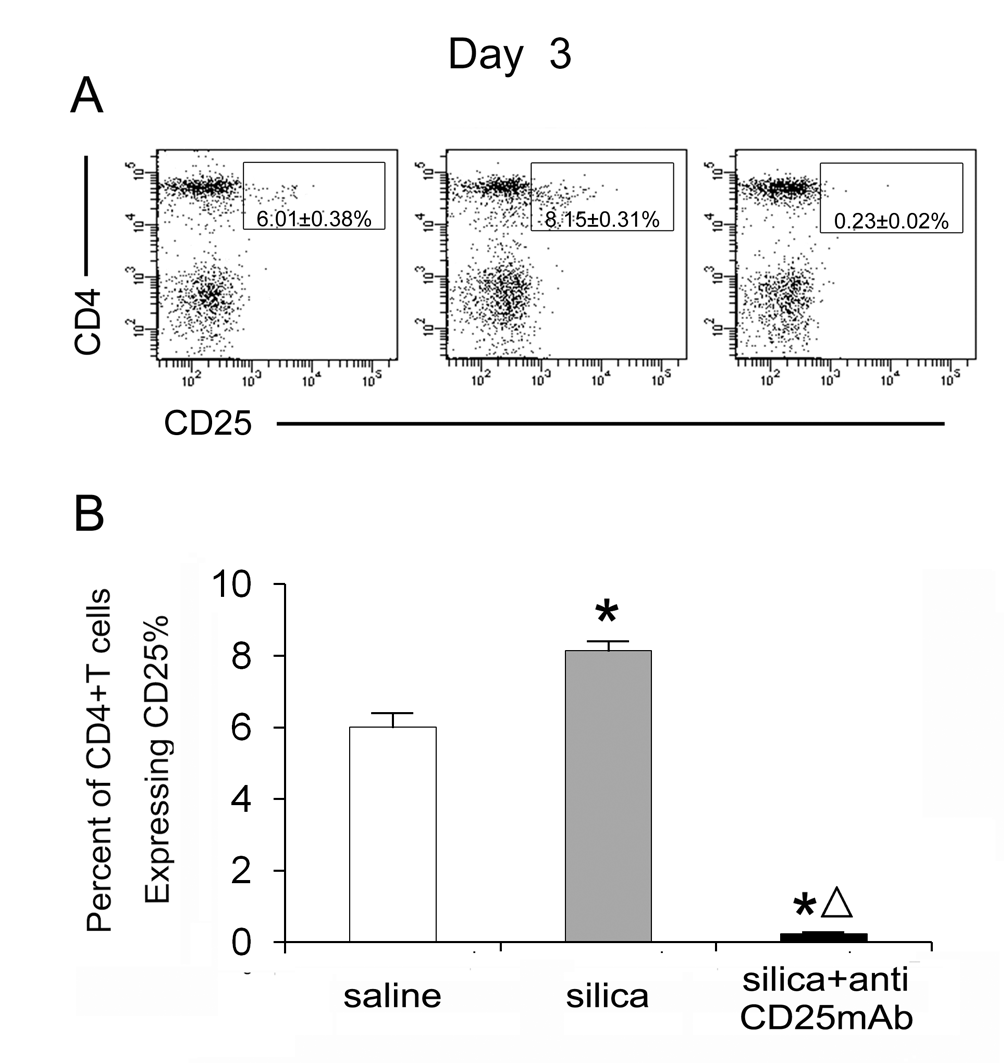

Supplement: Figure S1 — Injection of anti-CD25 mAb sufficiently depleted CD4+CD25+ regulatory T cells in vivo continuously. (A) C57BL/6 mice were treated i.p. with 100 µg anti-CD25 mAb or control IgG, the percentage of CD4+CD25+ Treg cells in the spleen was assayed by using anti-CD4 and CD25 mAb by flow cytometry (day 3). Results (n = 5) are shown as mean±SEM. (B) Percentage of CD4+ T cells expressing CD25 was shown in the graph (day 3). Results (n = 5) are shown as mean±SEM (one-way analysis of variance followed by pair-wise comparison with the Student-Newman-Keuls test. *, as compared with the saline control group, P<0.05; Δ, as compared with the silica group, P<0.05). (TIF) [file pone.0037286.s001.tif]

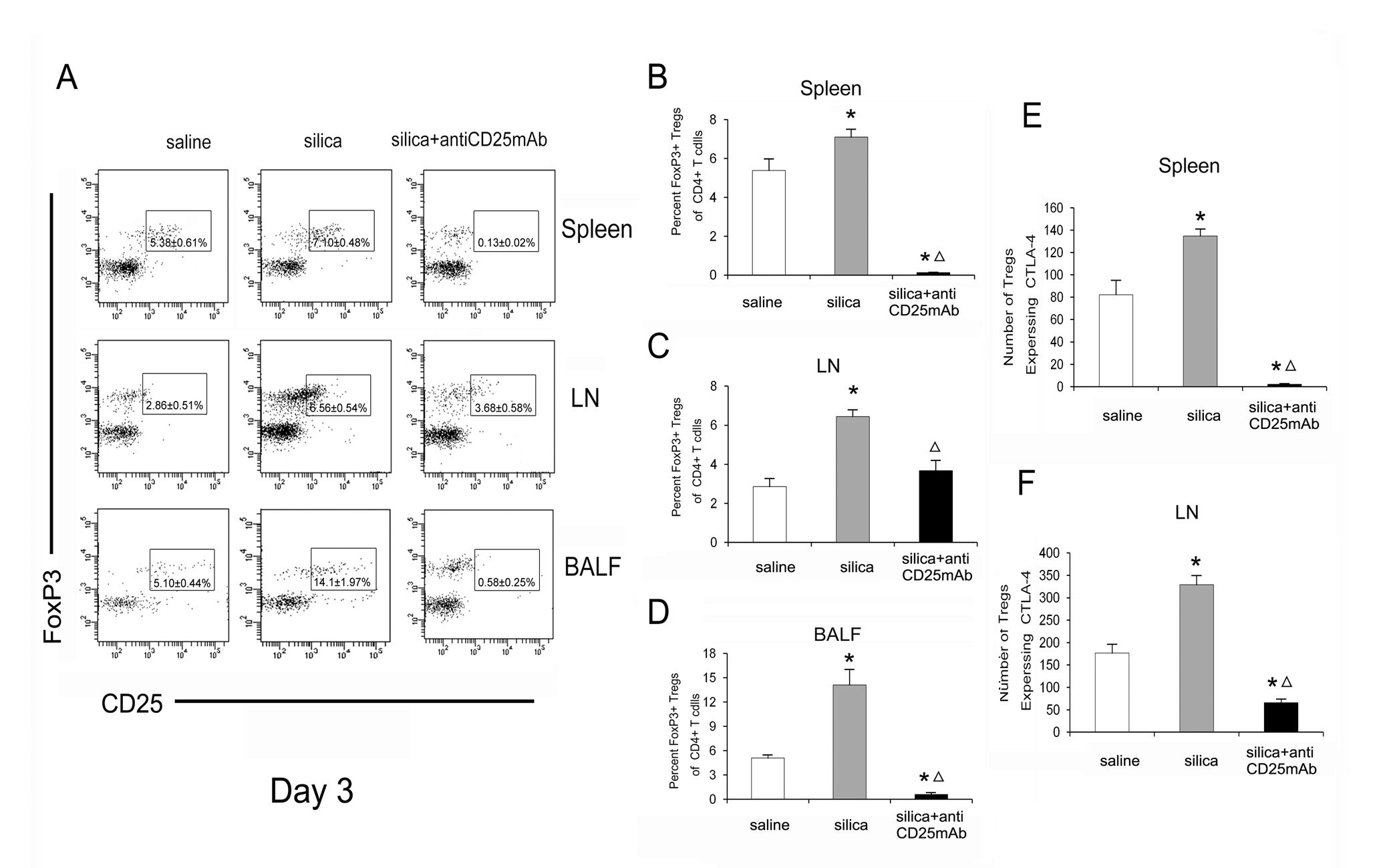

Supplement: Figure S2 — FOXP3 and CTLA-4, functional markers of Tregs, reduced clearly with the depletion of CD4+CD25+ Tregs. FOXP3+ Tregs in spleen, HLN, and BALF were calculated by flow cytometry (day 3) (A). The percentage of FOXP3+ Tregs was shown in the graph (B, spleen; C, HLN; D, BALF). The percentage of CTLA-4+ Tregs was shown in the graph (E, spleen; F, HLN). Results (n = 5) are shown as mean±SEM. (one-way analysis of variance followed by pair-wise comparison with the Student-Newman-Keuls test. *, as compared with the saline control group, P<0.05; Δ, as compared with the silica group, P<0.05). (TIF) [file pone.0037286.s002.tif]

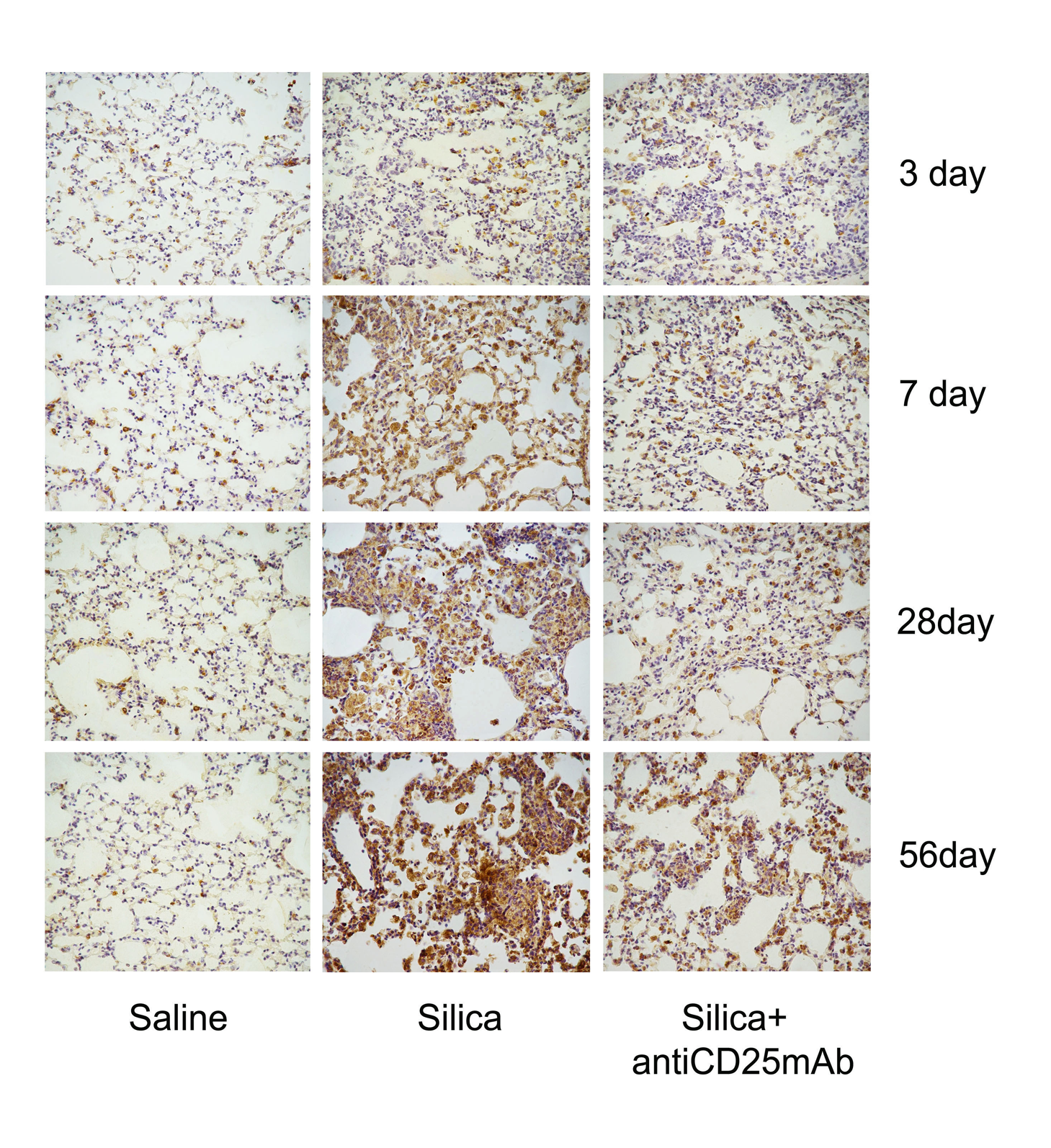

Supplement: Figure S3 — The protein level of TGF-β1 in mouse lungs examined by Immunohistochemistry (×200). Lung sections were examined by Immunohistochemistry. Positive staining is yellow brown, six random fields per sample (with n = 5 mice per group). (TIF) [file pone.0037286.s003.tif]

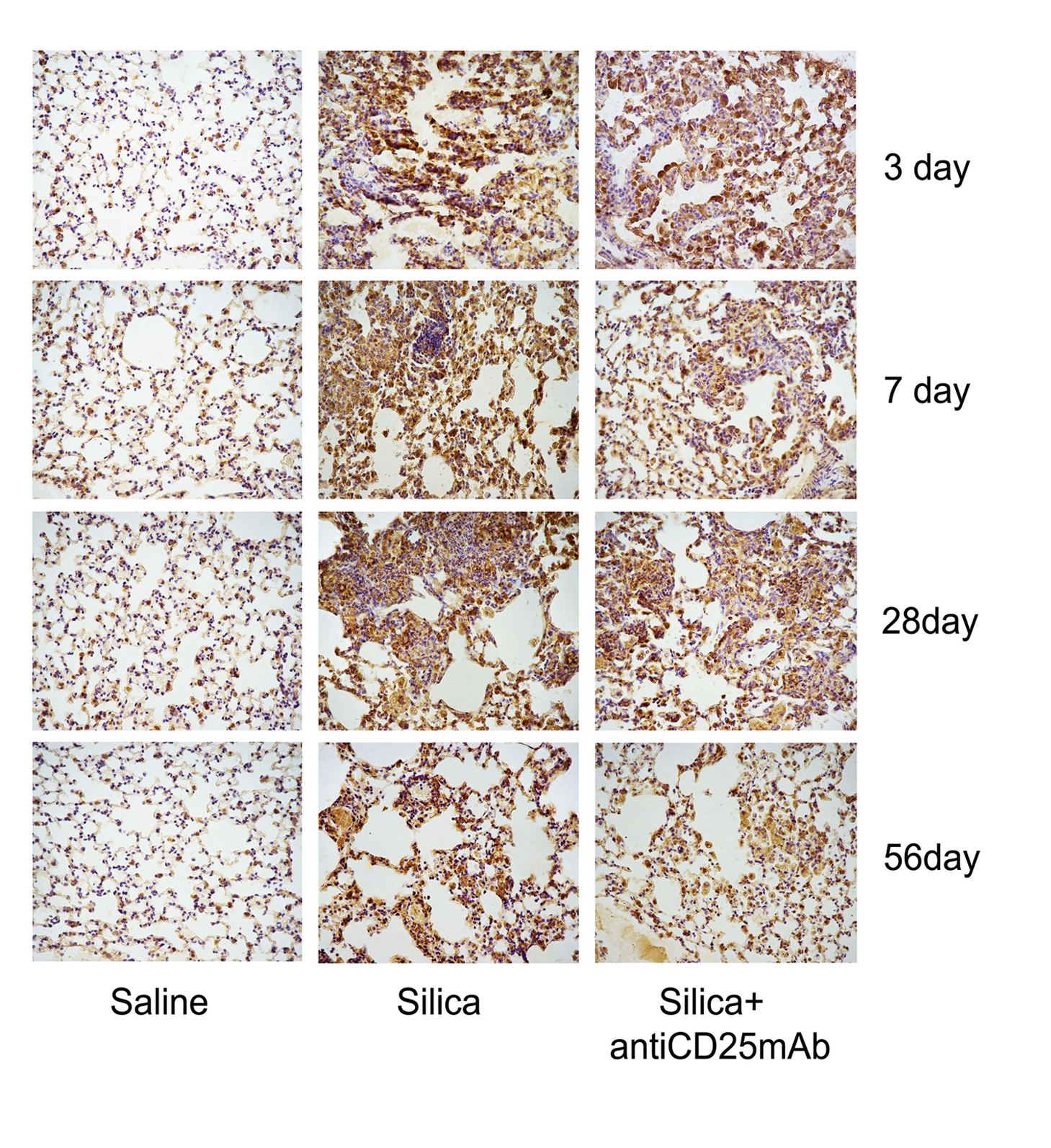

Supplement: Figure S4 — The protein level of IL-1β in mouse lungs examined by Immunohistochemistry (×200). Lung sections were examined by Immunohistochemistry. Positive staining is yellow brown, six random fields per sample (with n = 5 mice per group). (TIF) [file pone.0037286.s004.tif]

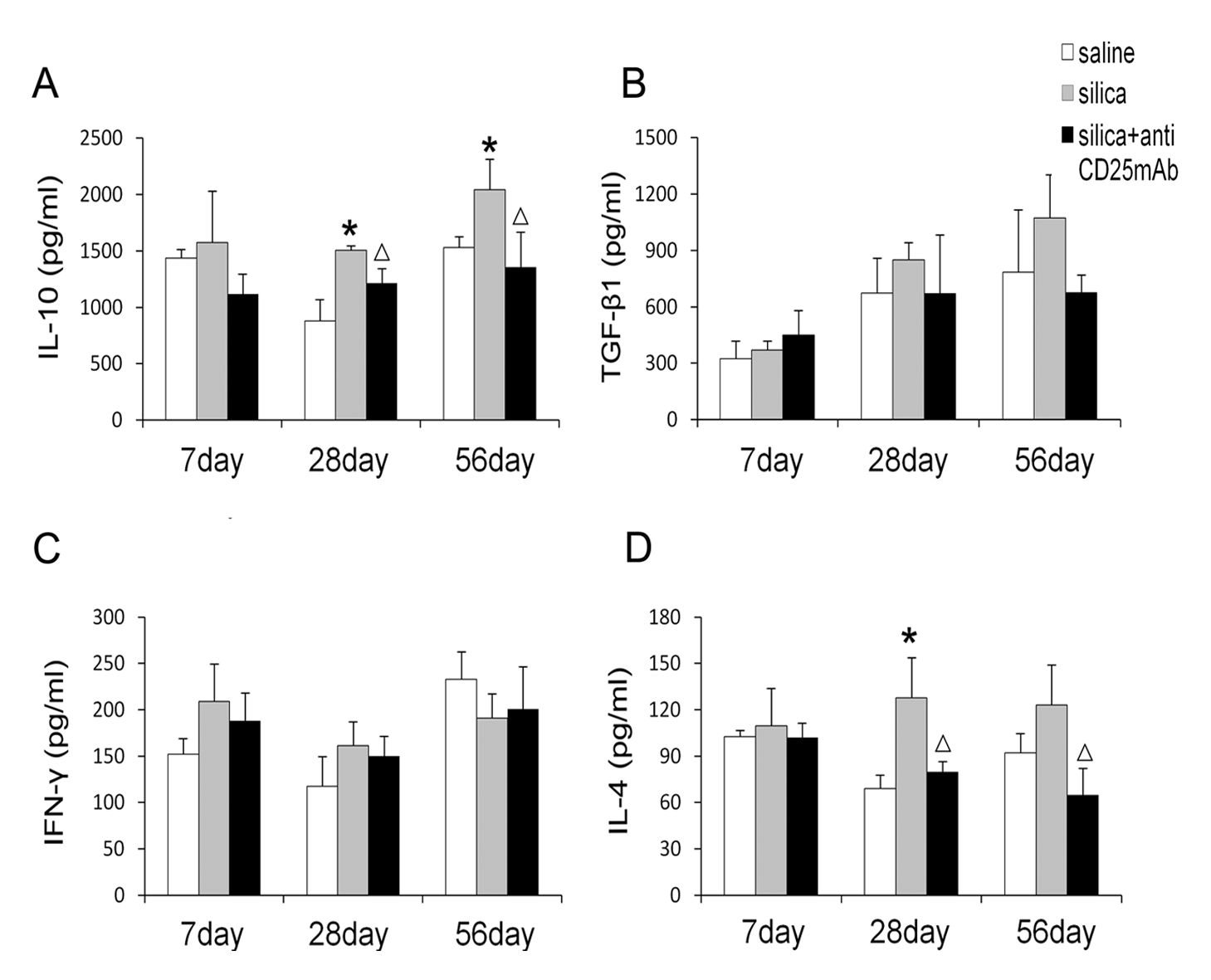

Supplement: Figure S5 — Treg cells affected the BALF cytokines in the mice model of silica-induced lung inammation. IL-10 (A), TGF-β1(B), Typical Th1 (IFN-γ)(C) and Th2 (IL-4)(D) cytokines in BALF were assayed by ELISA. (n = 3) (one-way analysis of variance followed by pair-wise comparison with the Student-Newman-Keuls test. *, as compared with the saline control group, P<0.05; Δ, as compared with the silica group, P<0.05). (TIF) [file pone.0037286.s005.tif]
